# Psychometric Properties of the “Alcohol Consumption Consequences Evaluation” (ACCE) Scale for Young Spanish University Students

**DOI:** 10.3389/fpsyg.2020.00649

**Published:** 2020-04-08

**Authors:** María-Dolores Sancerni-Beitia, José-Antonio Giménez-Costa, María-Teresa Cortés-Tomás

**Affiliations:** ^1^Department of Methodology of the Behavioral Sciences, Faculty of Psychology, University of Valencia, Valencia, Spain; ^2^Department of Basic Psychology, Faculty of Psychology, University of Valencia, Valencia, Spain

**Keywords:** alcohol drinking patterns, consequences, university students, psychometric properties, test validity

## Abstract

Instruments that evaluate alcohol use consequences among young people do not consider the intensive alcohol consumption pattern that is so characteristic during these ages. Some of these instruments are even ineffective in the Spanish population. Hence the interest in developing an instrument more adapted to the reality of our young people. A total of 601 university students (35.9% male and 64.1% female) from 18 to 20 years old were recruited. All of them answered a total of 77 items obtained from the review of both the scientific literature and the different scales used to measure consequences derived from alcohol consumption. In addition, they completed the AUDIT and the Timeline Followback for self-reported consumption. The data were analyzed using factor analysis and a two-parameter logistic model. ROC curve analysis was used to establish cut-off points for different risk levels of alcohol consumption distinguishing between genders. The final 43-item scale Alcohol Consumption Consequences Evaluation (ACCE) (Evaluación de Consecuencias derivadas del Consumo de Alcohol [ECCA]) shows adequate psychometric properties: α = 0.94; unidimensionality through exploratory factor analysis (EFA) (26.25% of explained variance) and confirmatory factor analysis (CFA) (RMSEA = 0.39; TLI and CFI > 0.90). In addition, ROC analyses, both at a global scale and distinguishing between genders, were able to characterize consumers with different levels of risk, obtaining areas under the curve between 0.82 and 0.88. A scale has been obtained that enables the establishment of cut-off points to distinguish between the consequences of low, moderate and high risk alcohol consumption. The clinical utility of the ACCE is highlighted by using one single instrument to perform the screening of a possible alcohol risk consumption as well as identifying the consequences that need to be worked on in the evaluated person’s or group’s intervention.

## Introduction

Alcohol consumption has been listed as a public health problem by being associated with a wide range of negative consequences (Hingson, 2010; Mason-Jones and Cabieses, 2015; De Bruyn et al., 2017; World Health Organization [WHO], 2018). Although people with dependency are more likely to experience damage due to their consumption, most of the problems associated with alcohol appear in non-dependent people (World Health Organization [WHO], 2001), especially among the young population (Cranford et al., 2006; Wechsler and Nelson, 2008).

In this last group, we must also highlight the high incidence of alcohol consumers. More specifically, in the Spanish population (Observatorio Español de las Drogas y las Adicciones [OEDA], 2017), 82.2% of young people between 18 and 20 years of age recognize they have consumed this substance during the last year, with a greater proportion of men (85.1%) than women (79%). A total of 46.3% of men and 32.5% of women have been reported to have had alcohol poisoning in the last 12 months and 37.3% and 29.41% of men and women, respectively, have engaged in intensive consumption or Binge Drinking (BD) during the last month.

In addition, the rate of university student consumers is higher compared to the incidence in non-university youths of the same age (Slutske, 2005; White et al., 2006; Carter et al., 2010). At an international level, approximately half of university students are involved in risky alcohol consumption patterns (Hingson, 2010; Moure-Rodríguez et al., 2014; Pilatti et al., 2017) which significantly increases the probability of experiencing negative consequences (Kuntsche et al., 2004; Martinotti et al., 2017). For example, in the United States 38% of university students aged 18–22 years reported carrying out risky consumption in the form of BD during the previous month, compared to 33% of their non-student peers (Substance Abuse and Mental Health Services Administration [SAMHSA], 2016) and in the United Kingdom, more than 60% of university students recognized engaging in BD (Cooke et al., 2007; Norman et al., 2012).

In this sense, there are several studies that try to identify the consequences derived from alcohol consumption in university students and the people surrounding them (Kahler et al., 2004; Hingson, 2010; Mallett et al., 2013; Motos, 2013; Hingson and White, 2014; Martinez et al., 2014; National Institute on Alcohol Abuse and Alcoholism [NIAAA], 2019) especially in regard to the consequences related to poor academic performance, memory gaps, interpersonal problems, adverse symptomatology associated with poisoning, risky or unplanned sexual behavior, longer reaction times and inhibition of driving ability, problems with justice, or causing inconvenience and harm to third parties, among others.

Given the important impact that alcohol consumption has at economic, social and health levels in young people (National Institute on Alcohol Abuse and Alcoholism [NIAAA], 2019), several international organizations (European Commission, 2006; World Health Organization [WHO], 2007) have endorsed for years the need to develop instruments for evaluating the consequences derived from this consumption behavior. This will allow for planning better-suited interventions which are better adjusted to the reality of this group.

Among the existing instruments used to evaluate alcohol consumption consequences, the following have been adapted and validated for the Spanish speaking population: *Rutgers Alcohol Problems Index (RAPI)* (White and Labouvie, 1989) adapted by López et al. (2012) and *Young Adult Alcohol Consequences Questionnaire (YAACQ)* (Kahler et al., 2005) adapted in Spanish by Pilatti et al. (2016) for a sample of young Argentinian nationals.

The first instrument (RAPI) has obtained good psychometric results in students between 16 and 18 years (López et al., 2012). However, being a tool created and validated with 12-, 15-, and 18-year-old adolescents (White and Labouvie, 1989), it only offers a partial view of the set of psychosocial consequences that young people may experience –including dependence, help-seeking and some adverse life consequences-. Certain important consequences for this population, such as driving under the influence of alcohol or lamenting having sex, are not contemplated in this instrument (Devos-Comby and Lange, 2008).

The second mentioned instrument (YAACQ) is a questionnaire which includes a continuum of consequences of differing severity that fall along a continuum from mild, relatively frequent consequences (e.g., headaches) to more sever, generally infrequent consequences (e.g., withdrawal symptoms (Pilatti et al., 2016). In addition, this instrument overcomes the gender-related bias of previous measures. In this case, externalized behaviors such as fighting or property damage, more characteristic of men (Crick and Zahn–Waxler, 2003), as well as internal consequences (e.g., decreased mood) and/or interpersonal (e.g., damaged relationships), more typical of women consumers, are included (Lo, 1996; Perkins, 2002).

Although YAACQ is generally supported in different countries as a measure of alcohol consequences in university students (Read et al., 2006, 2007; Pilatti et al., 2014; Keough et al., 2016), the study by Bravo et al. (2019) concludes that the findings are less solid in the Spanish sample, given that less consistent associations with consumption variables are appreciated, particularly those that include compliance reasons. In addition to all this, it should be noted that the external validity evidence of the YAACQ scores obtained by these same authors in a Spanish sample are not satisfactory. Additionally, the question of whether YAACQ works differently based on gender in different cultures remains unanswered.

Both the incidence of alcohol consumption in young people and the poor adequacy of the tools for assessing the consequences that are generated in this population sector justify the need to introduce new instruments that allow a more accurate assessment of the consequences associated with alcohol consumption in young Spaniards.

This article describes the psychometric properties of a measurement scale designed to evaluate a wide range of consequences associated with alcohol consumption in Spanish youth, both in males and females. As stated by Neal et al. (2006), Item Response Theory (IRT) offers an innovative method of scale development and validation for the measurements. Whereas more traditional psychometric methods (e.g., factor analysis, internal consistency) provide information regarding the performance of items in relation to the overall scale, IRT provides information about the performance of items in a theoretical underlying construct that the scale ostensibly measures.

Item response theory offers the possibility of evaluating the range of severity of the items. By examining the probability of acceptance of the items we know about their position in the trait (parameter *b*) and can therefore determine the items that have more severe levels of alcohol-related problems. In addition, through the discrimination parameter (parameter *a*) the minimum difference in the trait associated with a large difference in the probability of giving a response in one direction or another can be assessed, which indicates which items can better distinguish between people who are located at similar levels of the trait continuum. Finally, the IRT uses the information function to indicate the standard measurement error, denoting the accuracy with which the test measures along the different values of the scale of the measured variable; for each theta value a standard error value is attributed, so that SE is an indicator of precision (the smaller the value, the more precise).

To be more precise, the objectives of the present study are: (1) obtain evidence of validity based on the internal structure of the scale, (2) prove that the scale of consequences is useful for characterizing alcohol consumers with different levels of risk, and (3) evaluate the relationship of instrument scores with other theoretically relevant constructions of BD.

## Materials and Methods

### Participants

The sample of this study consists of 601 alcohol-consuming university students between 18 and 20 years old, with a mean age of 19.25 years (SD = 0.795). The main socio-demographic and alcohol consumption characteristics are shown in Table 1.

**TABLE 1:** Sample demographics and drinking characteristics: overall and by gender.

	Males	Females	Total	t	p
Participants	216 (35.9%)	385 (64.1%)	601		
Age (main, sd)	19.3	19.22	19.25	−1.26	0.207
	(sd. 0.79)	(sd. 0.79)	(sd. 0.79)		
Average age at which alcohol use started	15.36	14.90	15.07	3.77	>0.001
	(sd. 1.44)	(sd. 1.42)	(sd. 1.44)		
Engagement or not in BD	129 (59.7%)	255 (66.2%)	384 (63.9%)	*x*^2^ = 0.110	*x*^2^ = 0.112
Average SDUs when BD^a^	16.98	13.77	14.84	3.34	>0.001
	(sd. 9.30)	(sd. 8.60)	(sd. 8.96)		
Length of consumption when BD^a^	3.76	3.38	3.50	1.57	0.116
	(sd. 1.93)	(sd. 1.53)	(sd. 1.67)		
6-month frequency when BD^a^	9.29	8.11	8.51	1.19	0.234
	(sd. 9.64)	(sd. 7.93)	(sd. 8.55)		

*^*a*^Only for youths who engage in BD.*

### Procedure

The sample was recruited at the University of Valencia in two phases. In the first phase, the researchers visited first- and second-year Psychology students’ classrooms asking for student’s voluntary collaboration. Students who agreed to participate were summoned a day later to carry out the questionnaire. In a second phase, these participants were asked to make the research known among their classmates and provide the researchers’ contact information to classmates interested in participating.

For data collection, eight people received training in administering the instrument, so correct completion of it was guaranteed. All of them had two guided practices under the tutelage of the signatories of this study. Prior to the completion of the tests, all young people signed an informed consent, where the objectives of the investigation were clearly reflected, and the anonymity of the offered data was guaranteed.

The instrument was filled out in the presence of one of the interviewers.

The study was conducted in compliance with Spanish legislation (Ley Orgánica 3/2018, de 5 de diciembre) and the code of ethics for research involving human subjects, as outlined by the University of Valencia Human Research Ethics Committee. The survey used in this study is completely anonymous and there is no possibility of identifying the respondent. In addition, the survey itself includes an introduction that specifies the objectives to be achieved and the benefits it can bring, as well as an explicit reference to compliance with the current Data Protection Law. The last part of the introduction includes a paragraph in which the person indicates that they agree to participate voluntarily in the study. Due to missing information in some of the values of the instruments used, 57 questionnaires were discarded before entering the data. The use of any procedure to allocate deleted data was not contemplated, since a considerable sample size was already available, and it would have meant increasing the error size.

### Instruments

#### Consumption Pattern

The pattern of alcohol consumption was registered using a self-report in the format of a complete calendar of the last 6 months. This time interval makes it possible to account for the intermittent consumption made by young people, with periods of non-consumption even exceeding 30 days (Townshend and Duka, 2005; Courtney and Polich, 2009).

The self-report is an adaptation of the *Timeline Followback* (TLFB) by Sobell and Sobell (1996) and is used as a clinical and research method to obtain quantitative estimates of consumption. The calendar allows a person to record on a daily basis both frequency and amount of alcohol consumption as well as the moment at which consumption takes place. In other words, the start and end time of each intake are indicated, as well as the number of SBUs (Standard Beverage Unit) consumed. For the SBUs, participants were presented with a figure containing the equivalences between alcoholic beverages, their volume, and the number of respective SBUs.

From the information offered by the participants in the TLFB, the following variables were generated:

Maximum SBUs consumed: Of all the consumption episodes, the one with the highest amount of SBUs ingested was selected.

Engagement or not in BD: Based on the SDU consumed in the episode of maximum consumption and the number of hours in which this consumption took place, participants were classified as BD or non-BD. Following the most accepted definition in different reviews (Courtney and Polich, 2009; Parada et al., 2011; Cortés and Motos, 2015) in this study the proposal of the National Institute on Alcohol Abuse and Alcoholism [NIAAA] (2004) was used as a criterion to define BD, but in this case the grams of alcohol proposed by the original definition were adjusted to the Spanish SDU (1 SDU = 10 gr). Thus, men who consumed seven or more SDUs in a 2-h interval and six or more SDUs during the same time interval for females were classified as BD.

Frequency of BD: The days of BD in the last 6 months were added together.

### AUDIT

The only measures of alcohol consequences that have established cut-off scores to be used in the detection of alcohol consumption in university samples are the AUDIT (Babor et al., 2001) and its shorter version AUDIT C (Bush et al., 1998; Kokotailo et al., 2004; Adewuya, 2005; DeMartini and Carey, 2012). Both instruments have been applied in a variety of populations and can be used to detect the presence of alcohol-related problems. The AUDIT has also been used to evaluate evidence of validity for other instruments of alcohol use consequences in university students (De Bruyn et al., 2017).

The Spanish AUDIT validation (Cherpitel, 1995) was used in this study. This instrument consists of 10 items that measure risk consumption, dependency symptoms, and consequences associated with consumption, using a Likert response scale of five alternatives, ranging from 0 to 4, with the exception of items 9 and 10 that are evaluated with a Likert scale of three alternatives. In general, when the sum of the scores of all the items is eight or higher, the person is considered to undertake risky or harmful alcohol consumption. However, Cherpitel (1995) and Rosón (2008) differentiated the scores according to gender, identifying three consumer subtypes: low risk drinker (cut-off points of 0–7 in men and 0–5 in women), risk drinker (cut-off points of 8–12 and 6–12), and drinker with physical-psychic problems and probable alcohol dependence (cut-off point of 13 for both). This test has a good level of internal consistency, placing its reliability index at 0.88 (Guillamón et al., 1999). In this sample, the reliability coefficient was 0.78.

### Development of the Alcohol Consumption Consequences Evaluation -ACCE-

As a starting point for the development of this instrument, we proceeded to revise items included in different scales used to measure consequences derived from alcohol consumption in young people of different ages (YAACQ and RAPI) as well as other instruments of alcohol use aimed at the adult population that meet DSM criteria (*Drinker Inventory of Consequences DrInC*, Miller et al., 1995). We additionally identified consequences indicated by Cortés et al. (2015) considering the review of previous published research from the PsycARTICLES (ProQuest) database from the last 10 years, complementing it until 2017. A selection process was carried out to eliminate repeated consequences from different instruments, and to either combine those that showed complementary information or divide some that included more than one specific consequence in the same statement. Finally, a total of 77 items were collected.

This final combination of consequences included a broad spectrum of possible identifying alterations of the biopsychosocial spheres, both during consumption and in the short- and medium-term. In addition, this group of consequences also gathered some progress symptoms related to the addictive process which relates to loss of control, and possible signs of tolerance or withdrawal, all of them at different levels.

Initially, a 4-point Likert response scale was used (never; rarely; quite a few times; almost always), although later it was dichotomized, given that there were items in which the percentage of choice between alternatives was excessively small and therefore the difference between thresholds did not exist. In this way, 0 was assigned to the “never” category and 1 to all other categories. Several studies have assessed the psychometric properties of instruments in relation to the format used, concluding that the measurement of the instrument’s traits is not improved nor are the properties of the instrument affected by using a dichotomous or polytomous format (López Pina, 2005; Domínguez Lara, 2013). In addition, this dichotomization makes it possible to identify the different consequences present in a young person more accurately, considering that it is not the same to score with a high intensity in a certain consequence than to do so, even at a lower level, in several different consequences (Light et al., 2011).

Firstly, a frequency analysis was performed for the responses to the 77 items once these answers had been transformed into dichotomous variables. After the elimination of 17 items, due to their low frequency in any of the two categories, the sample was randomly divided into two subsamples (*n1* = 293; *n2* = 308) and tetrachoric correlations were used between the remaining 60 items to carry out factor analyses. With the first subsample, a maximum likelihood EFA with oblique rotation was performed, showing a KMO = 0.904 which supports the sample adequacy and a unifactorial solution that explained 26.25% of the variance compared to a second factor that only explained 5.32%. With the second subsample, a CFA was performed with an acceptable adjustment to the one-dimensional model (TLI = 0.918; CFI = 0.921; RMSA = 0.039; factor saturations between 0.52 and 0.79).

For the item selection, factor saturations greater than 0.50 and homogeneity indexes greater than 0.40 were established as criteria. There were 43 items that met both requirements.

With this solution, a CFA was applied using the entire sample in which a single factor was specified. The adequacy of the model in the CFA was checked with RMSEA with a value of 0.048, and the CFI and TLI indexes, which showed values of 0.925 and 0.921, respectively, exceeding the criteria of 0.90 recommended by Hu and Bentler (1999) to indicate the goodness of fit. Factor saturations ranged from 0.514 to 0.817 (Table 2). The residue analysis also let us conclude that the unidimensionality assumption was reasonably fulfilled, given that all residual correlations between the items were less than 0.10. Ordinal Cronbach’s alpha was 0.97.

**TABLE 2:** Item statistics and ITR parameters for the Alcohol Consumption Consequences Evaluation (ACCE).

Item	CFA loadings	*R*	Slope	Location	*z*-Resid	*p*
I have had a hangover the next morning after drinking	0.69	0.44	1.50	–1.36	0.66	0.51
I have felt tired and had less energy	0.65	0.46	1.46	–1.18	0.89	0.37
I drank in situations or moments I hadn’t planned to drink	0.60	0.44	1.35	–1.14	0.71	0.47
I was not able to wake up at my usual time	0.75	0.51	1.62	–0.94	0.83	0.40
I have had stomach aches and even vomited	0.67	0.50	1.55	–0.74	0.67	0.49
I drank more than I originally had planned to	0.77	0.55	1.86	–0.73	0.38	0.70
I have come to wobble or fall	0.71	0.53	1.66	–0.58	0.61	0.54
I haven’t been as agile mentally	0.67	0.50	1.50	–0.57	0.83	0.40
I drank for a longer period of time than I intended to	0.75	0.57	1.89	–0.53	0.63	0.53
I have been in the car with a driver under the influence of alcohol	0.51	0.38	1.03	–0.48	0.59	0.55
I have been less active physically	0.69	0.52	1.58	–0.38	1.15	0.24
I have become clumsy	0.59	0.44	1.26	–0.37	0.90	0.36
I haven’t eaten properly	0.64	0.50	1.48	–0.35	1.23	0.22
I have said or done embarrassing things	0.81	0.64	2.40	–0.20	0.65	0.51
I have had a hard time	0.65	0.52	1.56	–0.18	1.28	0.20
I have felt guilty or embarrassed	0.69	0.53	1.66	–0.05	0.72	0.47
I have been “forgetful”	0.72	0.57	1.89	–0.03	0.57	0.56
I have done impulsive things I later regretted	0.81	0.65	2.50	–0.02	0.93	0.35
I have felt bad about myself	0.64	0.49	1.47	0.12	0.93	0.35
I haven’t slept properly	0.53	0.41	1.16	0.17	1.12	0.26
I have said harsh or cruel things	0.64	0.49	1.49	0.20	0.85	0.39
I have realized I now need more alcohol than some years ago to notice its effects or to get drunk	0.69	0.53	1.70	0.24	0.52	0.59
My condition after drinking has resulted in lower work or study performance in comparison to days without previous drinking	0.62	0.48	1.44	0.25	1.37	0.16
I might have caused someone else to feel embarrassed	0.71	0.56	1.92	0.26	0.35	0.72
I have had less time to do my activities or entertain myself	0.67	0.53	1.71	0.29	1.22	0.22
I smoke more when I drink or I only smoke when I drink	0.66	0.52	1.67	0.35	1.01	0.31
Once I start, I find it difficult to know when I should stop drinking	0.70	0.54	1.79	0.36	1.28	0.20
When I drink excessively, I am not able to remember what happened during large periods of time	0.72	0.56	1.98	0.42	0.52	0.60
I have felt disappointed with myself	0.68	0.51	1.73	0.52	0.44	0.65
My physical appearance has been negatively affected by my drinking	0.66	0.51	1.71	0.54	0.45	0.65
I have noticed a change in my personality	0.55	0.41	1.24	0.56	0.50	0.61
I have had conflicts or arguments with people who are close to me	0.72	0.55	2.08	0.59	0.30	0.76
I have even felt sad or depressed	0.66	0.51	1.75	0.61	0.49	0.62
I have become very rude, unpleasant or offensive when drinking	0.72	0.52	1.93	0.73	0.47	0.63
I have occasionally felt the need to consume alcohol	0.59	0.45	1.52	0.86	0.52	0.60
I have stopped going to work or I have missed classes because I felt unwell	0.64	0.47	1.72	0.87	1.16	0.24
My relationship with someone close to me has been affected	0.65	0.48	1.79	0.89	0.85	0.39
I have suddenly found myself in a place unable to remember how I got there	0.58	0.43	1.46	0.91	0.40	0.68
I have come to feel unhappy	0.61	0.44	1.66	1.02	0.77	0.44
I have missed out on some things because I spent too much money on alcohol	0.63	0.46	1.94	1.14	0.93	0.35
I have neglected my responsibilities with family, work, or studies	0.69	0.47	2.07	1.16	0.88	0.37
When I’m drinking, I take other drugs that I don’t normally consume at other times	0.56	0.40	1.42	1.21	0.68	0.49
My parents, my partner or a friend have asked me to stop drinking or to drink less	0.54	0.38	1.50	1.37	0.71	0.48

*Items are sorted by item severity. CFA loadings, factor loading in CFA. R, multiple correlation. Slope, slope (discrimination) parameter estimates; Location, location (severity) parameter estimates. Statistic fit of the items: *z*-resid and *p*.*

### Data Analysis

Statistical analyses were carried out with IBM-SPSS Statistics 22, MPlus 7.4, and XCalibre 4.1.8.

To assess the evidence of validity based on the internal structure of the scale, the double strategy used by Van Dam et al. (2010) and Wang and Russell (2005) was applied: exploratory factor analysis (EFA) and confirmatory factor analysis (CFA) combined with models of the IRT.

The sample was randomly divided into two subsamples (*n1* = 293; *n2* = 308). A maximum likelihood EFA with oblique rotation was performed with the first sample, using the criterion of considering the unifactorial scale if the first factor exceeds 25% of the explained variance. CFA was used for the second sample using chi-square indexes, Root Mean Square Error of Approximation (RMSEA), Comparative Fit Index (CFI) and Tucker-Lewis Index (TLI) to evaluate the fit, with the following cut-off points: RMSEA < 0.05; CFI and TLI > 0.90; and factorial saturations > 0.50.

Several studies on alcohol-related consequences have used the IRT and have shown its usefulness (Kahler et al., 2004, 2005; Neal et al., 2006). The IRT provides a method to develop a scale and obtain evidence of validity to assess the execution of the items in relation to a theoretical construct that measures the scale. In this case, a two-parameter model was used: that of severity or position and that of discrimination. With the first one, information is obtained regarding the items’ order, so that the probability of being at various trait levels can be interpreted as the items being indicators of different levels of alcohol-related problems. The second parameter allows us to assess small trait divergences associated with a large difference in the item’s acceptance probability, which indicates the item works well by placing an individual at a specific point.

The Mantel-Haenszel (MH) procedure was used for the detection of the item’s uniform differential functioning according to gender. DIF enables the assessment of whether the location (b) and slope (a) parameters for each item vary regarding gender. This analysis assesses whether the proportion of choice of the item is equal in young males and young females for those young people who have a similar level of the measured trait.

Excessive alcohol consumption and BD are related to the negative consequences that students experience (Wechsler et al., 1994; De Bruyn et al., 2017). Therefore, and to prove the scale’s usefulness to characterize alcohol consumers with different levels of risk, a ROC curve analysis was performed, using the scores in the AUDIT questionnaire as criteria. This analysis was carried out in two phases, both with the complete sample and distinguishing between genders. In the first phase, we sought to distinguish between low and moderate-high risk and in the second phase between moderate-high and high risk. The Youden index (J = specificity + sensitivity − 1) was also calculated for the selection of cut-off points together with the sensitivity value. The appropriate *t*-tests were also performed according to gender and BD.

Finally, to account for the last specific objective, a correlation analysis was carried out with frequency and intensity measurements of BD.

## Results

Due to the fact that the final scale of 43 items was one-dimensional, which is a requirement to use a TRI model, a two-parameter logistic model was applied, obtaining a fit (Chi-square_(559)_ = 592.097; *p* = 0.161). The discrimination values and severity parameters are shown in Table 2, ordered according to the severity parameter.

The order of the items presented in Table 2 reflects a continuum of alcohol-related problems. The severity parameter identifies what consequences warn of problems of special relevance to be considered in terms of prevention/intervention, given that to score on items whose severity is high, more trait is required. The discrimination parameter, which indicates that the small divergences in the trait are associated with a large difference in the probability of item acceptance (showing that the item works well by placing an individual at a specific point), reports that the most discriminating items are those related to impulsivity behavior and relationships with those close to you. All items exceed the minimum threshold established by Baker and Kim (2004) between 1.03 and 2.50.

The estimated trait for each of the participants through the applied model was examined for each total score on the scale. As expected, the estimated average score on the trait increases as the score on the scale increases, although that increase is not linear.

**FIGURE 1:**
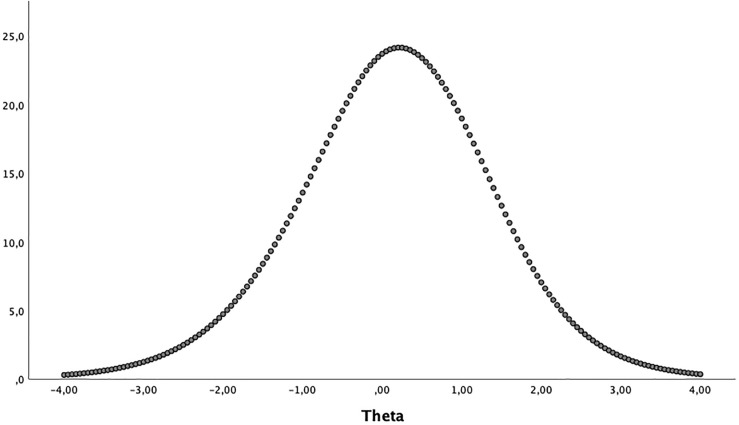
Test’s information function. This figure displays a graph of the Conditional Standard Error of Measurement (CSEM) Function. The CSEM is an inverted function of the TIF, and estimates the amount of error in theta estimation for each level of theta. The minimum CSEM was 0.203 at theta = 0.200.

The curve of the test’s information function shows the measurement accuracy along with the trait. It has its maximum at 0.20 (Figure 1) equivalent to a score of 24 points, which corresponds to the middle area of the trait continuum, indicating that the scale is better for estimating the trait of young people who are close to that maximum.

Additionally, we analyzed the possibility of uniform differential item functioning (DIF) according to gender, through the MH method. It was not significant in any item. The comparison of means was not significant either (*t* = 0.94; *p* = 0.347), the average of the females being 20.59 (SD = 10.60), and that of the males being 19.74 (SD = 10.86). The effect size was *d* = 0.08.

Evidence of validity to prove that this scale can characterize alcohol consumers with different risk levels was obtained using ROC curves with the AUDIT scores as criteria. In this way, the scores that showed greater sensitivity were established and used to classify young people in groups of different risk levels with a high percentage of success. This analysis was carried out with the complete sample and distinguishing between gender in the same way as Read et al. (2016).

The analysis was carried out in two phases. In the first, the cut-off points sought to distinguish between low vs. moderate-high risk. The result of the ROC analysis presents an area of 0.839 (SE = 0.016) for the complete sample with a 95% confidence interval between 0.807 and 0.871. In the case of females, the area was 0.851 (SE = 0.019) with a 95% confidence interval between 0.813 and 0.889. Finally, for males, the area was 0.826 (SE = 0.030) with a 95% confidence interval between 0.767 and 0.884.

In the second phase, the cut-off points sought to distinguish between low-moderate vs. high risk. Again, the analyses were carried out with the complete sample and distinguishing between genders. The ROC analysis offered an area for the entire sample of 0.856 (SE = 0.023) with a 95% confidence interval between 0.811 and 0.900. For females, the area was 0.887 (SE = 0.023) with a 95% confidence interval between 0.843 and 0.932. Finally, for males, the area was 0.836 (SE = 0.037) with a 95% confidence interval between 0.763 and 0.908.

**FIGURE 2:**
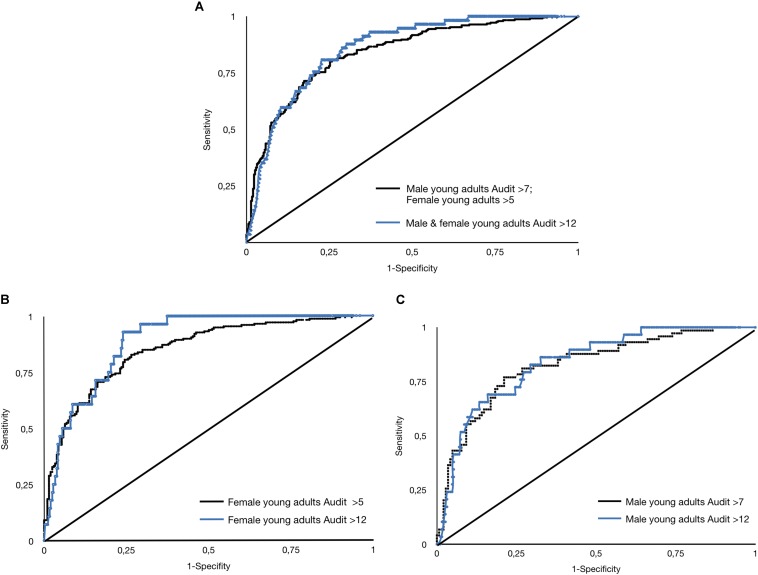
ROC curves comparing AUDIT cut-off values. **(A)** Distinction between low vs. moderate-high risk, including male and female young adults; **(B)** Distinction between low vs. moderate-high risk, only for female young adults; **(C)** Distinction between low vs. moderate-high risk, only for male young adults.

The ROC curves obtained are shown in Figure 2.

Table 3 shows the sensitivity, specificity and Youden index values for each raw score and its corresponding θ score. Although in this study we chose to select the cut-off points that maximize sensitivity, an agreement can be observed in the case of low vs. moderate-high risk comparison with the values recommended by the Youden index and the value selected according to sensitivity for the total sample and females. Given that the objective of this scale is to verify its usefulness as an instrument for preventing the development of potential addictive behavior, prioritizing the sensitivity, which means prioritizing the probability of detecting the problem when it is present, was considered more appropriate. This led to a cut-off score of 20. In the case of the low-moderate vs. high risk comparison, the value of the cut-off point on the scale rises to 24.

**TABLE 3:** Summary of the statistics for the receiver operating characteristic (ROC) analysis to determine cut-off values for the Alcohol Consumption Consequences Evaluation (ACCE).

	Total Sample				Females Only				Males Only			
	AUDIT cut-off value >5 for				AUDIT cut-off value >5				AUDIT cut-off value >7			
	females and >7 for males													
Score	M	Sensitivity	Specificity	Youden’s Index	*M*	Sensitivity	Specificity	Youden’s Index	*M*	Sensitivity	Specificity	Youden’s Index
15	–0.28	0.91	0.50	0.41	–0.30	0.92	0.52	0.44	–0.25	0.89	0.46	0.35
16	–0.24	0.90	0.51	0.411	–0.24	0.91	0.55	0.46	–0.25	0.89	0.46	0.35
17	–0.17	0.88	0.57	0.45	–0.17	0.89	0.61	0.50	–0.25	0.89	0.46	0.35
18	–0.05	0.86	0.64	0.50	–0.06	0.85	0.67	0.52	–0.04	0.86	0.61	0.47
19	–0.04	0.85	0.65	0.50	–0.06	0.85	0.67	0.52	0.01	0.83	0.62	0.45
20	0.03	0.83	0.69	0.52	0.04	0.83	0.72	0.55	0.01	0.83	0.62	0.45
21	0.13	0.81	0.73	0.54	0.13	0.80	0.76	0.56	0.14	0.81	0.72	0.53
22	0.18	0.77	0.76	0.53	0.19	0.76	0.77	0.53	0.18	0.78	0.75	0.53
23	0.29	0.71	0.82	0.53	0.30	0.71	0.84	0.55	0.27	0.74	0.79	0.53
24	0.32	0.69	0.84	0.53	0.32	0.68	0.85	0.53	0.34	0.71	0.82	0.53
25	0.39	0.63	0.85	0.48	0.40	0.61	0.87	0.48	0.38	0.67	0.83	0.50
26	0.48	0.58	0.88	0.46	0.49	0.57	0.90	0.47	0.43	0.62	0.84	0.46
27	0.56	0.54	0.91	0.45	0.57	0.52	0.93	0.45	0.56	0.56	0.90	0.46
28	0.62	0.51	0.93	0.44	0.61	0.51	0.93	0.44	0.69	0.50	0.91	0.41
29	0.70	0.47	0.93	0.40	0.71	0.45	0.95	0.40	0.71	0.47	0.91	0.38
30	0.76	0.42	0.95	0.37	0.76	0.41	0.96	0.37	0.74	0.45	0.93	0.38

	**AUDIT cutoff value >12**											
**Score**	**M**	**Sensitivity**	**Specificity**	**Youden’s Index**	***M***	**Sensitivity**	**Specificity**	**Youden’s Index**	***M***	**Sensitivity**	**Specificity**	**Youden’s Index**

15	–0.18	0.98	0.40	0.38	–	1	0	0	–0.18	0.93	0.42	0.35
16	–0.18	0.98	0.40	0.38	–	1	0	0	–0.18	0.93	0.42	0.35
17	–0.18	0.98	0.40	0.38	–	1	0	0	–0.18	0.93	0.42	0.35
18	–0.18	0.98	0.40	0.38	–	1	0	0	–0.18	0.93	0.42	0.35
19	0.01	0.95	0.50	0.45	–	1	0	0	0.01	0.89	0.52	0.41
20	0.01	0.95	0.50	0.45	–	1	0	0	0.01	0.89	0.52	0.41
21	0.12	0.94	0.55	0.49	–	1	0	0	0.12	0.88	0.59	0.47
22	0.12	0.94	0.55	0.49	–	1	0	0	0.12	0.88	0.59	0.47
23	0.29	0.91	0.65	0.56	0.31	0.96	0.63	0.59	0.28	0.82	0.68	0.50
24	0.34	0.89	0.68	0.57	0.31	0.96	0.63	0.59	0.34	0.82	0.69	0.51
25	0.41	0.86	0.71	0.57	0.43	0.96	0.71	0.67	0.39	0.76	0.73	0.49
26	0.43	0.84	0.72	0.56	0.43	0.96	0.71	0.67	0.43	0.72	0.75	0.47
27	0.57	0.77	0.78	0.55	0.57	0.89	0.76	0.65	0.43	0.72	0.75	0.47
28	0.61	0.75	0.79	0.54	0.61	0.82	0.77	0.59	0.43	0.72	0.75	0.47
29	0.69	0.73	0.81	0.54	0.70	0.78	0.80	0.58	0.69	0.69	0.84	0.53
30	0.75	0.86	0.84	0.52	0.74	0.71	0.81	0.52	0.77	0.65	0.87	0.52

**M*, average estimated latent-trait score for the raw score on the ACCE.*

As seen in Table 3, there is a greater increase in the value of the trait when the established cut-off scores of 20 and 24 are reached. Thus, the total scores on the presented scale can be used to classify young people into three levels of risk based on the ROC analysis. Firstly, a low risk level of alcohol consumption would comprise people whose scores are below 20 (*N* = 292; 48.6%). Secondly, scores between 21 and 24 would correspond to a moderate risk level (*N* = 86; 14.3%). Third and finally, scores above 25 would indicate high risk levels (*N* = 223; 37.1%).

Despite not finding large differences between male and female, differences according to gender were checked in each of these levels. At the low level, no differences were obtained (*t* = 0.676; *p* = 0.499), the average being 11.34 (SD = 6.168) for females and 10.83 (SD = 6.436) for males. The size of the effect was *d* = 0.083. At the moderate level, differences did appear (*t* = 2,425; *p* = 0.017), with the average of females being 22.78 (SD = 1.155) and that of males 22.18 (SD = 0.863). The size of the effect was *d* = 0.517. At the high level, no differences were obtained (*t* = 0.464; *p* = 0.643), the average being 31.47 (SD = 4.726) and 31.16 (SD = 4.741) for females and males, respectively. The size of the effect was *d* = 0.065.

Table 4 shows the estimated latent-trait scores for the ACCE by each score of the 43 items. The latent-trait scores increase as total score on the ACCE increases. However, the increase does not occur linearly, increases of 1 in the total ACCE score do not correspond to an equivalent increase in the latent-trait score across the entire range of ACCE scores.

**TABLE 4:** Correspondence of the ACCE scores to latent trait scores.

ACCE score	Theta	CSEM	ACCE score	Theta	CSEM
0	–3.15	0.99	22	0.25	0.20
1	–2.35	0.57	–23	0.30	0.20
2	–1.95	0.44	24	0.35	0.20
3	–1.70	0.38	25	0.45	0.20
4	–1.50	0.34	26	0.50	0.20
5	–1.30	0.31	27	0.60	0.21
6	–1.15	0.29	28	0.65	0.21
7	–1.05	0.27	29	0.75	0.21
8	–0.90	0.26	30	0.85	0.22
9	–0.80	0.25	31	0.90	0.22
10	–0.70	0.24	32	1.00	0.23
11	–0.60	0.23	33	1.10	0.23
12	–0.50	0.22	34	1.20	0.24
13	–0.45	0.22	35	1.30	0.25
14	–0.35	0.21	36	1.45	0.27
15	–0.25	0.21	37	1.55	0.28
16	–0.20	0.21	38	1.70	0.31
17	–0.10	0.20	39	1.90	0.35
18	–0.05	0.20	40	2.15	0.41
19	0.05	0.20	41	2.50	0.53
20	0.10	0.20	42	3.25	0.94
21	0.15	0.20	43	3.90	1.55

*Theta = estimated latent-trait score for this raw score on the ACCE; CSEM = Conditional standard error of measurement.*

In addition, the correlation of the theta score on this scale of consequences was calculated with two indicators of intensity (maximum SBUs consumed) and frequency (BD frequency), correlations that turned out to be significant, 0.14 (*p* = 0.007) and 0.36 (*p* = 0.000), respectively.

When comparing the means on the scale according to the variable “Engagement or not in BD,” significant differences were obtained as expected (*t* = 10.88; *p* = 0.000), the average being the highest in the consequence’s scale for those engaging in BD (23.55, SD = 9.502) as opposed to those not engaging (14.51; SD = 10.263). The size of the effect was *d* = 0.95.

## Discussion

This article describes the psychometric properties of a measurement scale designed to evaluate a wide range of consequences associated with alcohol consumption in Spanish university students. To do so, the first step consisted of developing a set of consequences that would overcome the problems shown by similar scales developed in other countries. The final instrument (ACCE) integrated consequences included partially or in their entirety in other scales, although with different wording, and 10 next-generation elements resulting from the review of the aforementioned literature.

Three specific objectives were formulated to obtain evidence that supported the adequacy of the presented questionnaire. The first one focused on obtaining evidence about the internal structure of the instrument. The double procedure used to calculate the scale properties, factor analysis, and TRI, show good results, obtaining a maximally useful one-dimensional scale at the average risk levels of alcohol consumption. By doing this, an instrument is obtained that enables the evaluation of a heterogeneous range of consequences which are part of a single continuum. Having achieved a set of items which can be adjusted to a two-parameter logistic model has allowed ordering the different consequences within this continuum, as well as detecting those which can be discriminating. For example, “saying shameful things” or “doing impulsive things with subsequent regret” are items that, with greater visibility, identify subjects with a low level of severity. While “having conflicts with close people” or “neglecting responsibilities with family, work or studies” stand out for being items that clearly identify a high level of severity.

The second specific objective focused on the search for additional evidence on the usefulness of the scale to characterize alcohol consumers with different risk levels. More specifically, the indicator items of low risk levels (scores below 20) include physical consequences of excessive consumption (hangover, clumsiness). At the moderate level of risk (between 21 and 24 point), the indicators reflect social disruption such as being cruel, causing shame and lack of performance. The consequences grouped in the high risk level (scores higher than 25) are indicative of more serious or disruptive problems, such as isolation behavior, sadness or concern of friends and family in relation to the consumption.

These results support the value of ACCE as a scale that allows professionals to identify the greater or lesser urgency in the implementation of necessary measures to slow the progression of young adults within the addictive process.

The ACCE covers one of the shortcomings noted by Falk et al. (2010) and Kirouac and Witkiewitz (2018) in regard to analyzing the utility of consequences thorough psychological work as a sensitive measure over indicators of quantity of consumption while preforming interventions.

Another important aspect of this scale, since DIF is not found according to gender in any of the elements, is that it can use the same elements and cut-off points with all young adults.

The third and final specific objective was to evaluate the relationship between ACCE scores and consumption patterns which are characteristic of the university population, as is the case of BD. The results show that this instrument presents a high relationship with the act of engaging or not in BD, as well as with defining parameters of this pattern such as the amount of SBUs consumed (intensity) and the frequency of engagement in BD. It can be said that the instrument that identifies the relevant consequences of a consumption pattern as characteristic of young adults as is the BD (Substance Abuse and Mental Health Services Administration [SAMHSA], 2016). The instrument can be said to be able to identify relevant consequences of consumption patterns in young adults as characteristic as BD (Substance Abuse and Mental Health Services Administration [SAMHSA], 2016).

The progression of the ACCE items goes from items with which a greater number of young people are identified (extracted from the literature review and other similar ones to those included in the YAAQC), toward another set of elements identified in clinical populations. As a result, the new instrument offers a more comprehensive picture of the consequences generated by the alcohol consumption of male and female young adults, reflecting the bio-psychosocial aspects of the whole addictive process, and therefore showing the wide variability of existing consumers.

In conclusion, ACCE shows its clinical worthiness by facilitating the identification of young people whose behavior toward alcohol use could qualify as risky, using the consequences that they themselves have experienced when consuming alcohol. Accurate detection of risk-taking behaviors through routine examinations in university settings could help identify students at risk, raise awareness of the need for change, and connect them with the most appropriate interventions to meet their specific needs (Read et al., 2016).

Finally, we must point out the limitations of this study. Although the recruitment of volunteers tried to ensure a balanced sample between genders, the imbalance in the proportion between them is evident. However, it is even smaller than in other studies of instrument validation for alcohol in university students (Scully et al., 2018). To address these limitations, future research should try to replicate the findings of the present study with larger group sizes and with a more equitable gender distribution, even in different regions of the country. This improvement in the sample would support the clinical utility of the ACCE and would confirm the differences between males and females in the number of consequences, especially within the moderate risk level.

The replication of the results of this study in university students from other countries with similar alcohol consumption patterns would allow generalizing the usefulness of the ACCE as a research instrument and as a predictive and identifying measure of problems associated with alcohol consumption. This generalization would also be extended if samples of non-university youth of similar ages were included. In future research, it would also be interesting to control for variables that could mask or enhance some of the problems such as some psychopathologies (symptoms of depression or anxiety) or the consumption of medications.

## Data Availability Statement

The datasets generated for this study are available on request to the corresponding author.

## Ethics Statement

Ethical review and approval was not required for the study on human participants in accordance with the local legislation and institutional requirements. The patients/participants provided their written informed consent to participate in this study.

## Author Contributions

All authors designed and carried out the study, registered, collected and analyzed all the data presented, and also wrote both the theoretical and experimental part of the manuscript.

## Conflict of Interest

The authors declare that the research was conducted in the absence of any commercial or financial relationships that could be construed as a potential conflict of interest.

## References

[B1] Adewuya A. O. (2005). Validation of the alcohol use disorders identification test (AUDIT) as a screening tool for alcohol-related problems among Nigerian university students. *Alcohol Alcohol.* 40 575–577. 10.1093/alcalc/agh197 16115823

[B2] Babor T. Higgins-Biddle J. Saunders J. Monteiro M. (2001). *AUDIT: The Alcohol Use Disorders Identification Test, Guidelines for Primary Care*, 2nd Edn Geneva: World Health Organization.

[B3] Baker F. B. Kim S. H. (2004). *Item Response Theory Parameter Estimation Techniques*, 2nd Edn Boca Raton, FL: CRC Press.

[B4] Bravo A. J. Pilatti A. Pearson M. R. Read J. P. Mezquita L. Ibáñez M. I. Ortet G. (2019). Cross-cultural examination of negative alcohol-related consequences: measurement invariance of the young adult alcohol consequences questionnaire in Spain, Argentina, and USA. *Psychol. Assess.* 31. 631–642. 10.1037/pas0000689 30667265 PMC6488382

[B5] Bush K. Kivlahan D. R. McDonell M. B. Fihn S. D. Bradley K. A. (1998). The AUDIT alcohol consumption questions (AUDIT-C): an effective brief screening test for problem drinking. *Arch. Intern. Med.* 158 1789–1795. 10.1001/archinte.158.16.1789 9738608

[B6] Carter A. C. Brandon K. O. Goldman M. S. (2010). The college and noncollege experience: a review of the factors that influence drinking behavior in young adulthood. *J. Stud. Alcohol. Drugs* 71 742–750. 10.15288/jsad.2010.71.742 20731981 PMC2930506

[B7] Cherpitel C. J. (1995). Analysis of cut points for screening instruments for alcohol problems in the emergency room. *J. Stud. Alcohol.* 56 695–700. 10.15288/jsa.1995.56.695 8558901

[B8] Cooke R. Sniehotta F. Schuez B. (2007). Predicting binge-drinking behaviour using an extended TPB: examining the impact of anticipated regret and descriptive norms. *Alcohol Alcohol.* 42 84–91. 10.1093/alcalc/agl115 17185302

[B9] Cortés M. T. Motos P. (2015). “Cómo definir y medir el consumo intensivo de alcohol,” in *Guía Clínica. Consumo Intensivo de Alcohol en Jóvenes*, ed. Cortés (Barcelona: Socidrogalcohol), 25–46.

[B10] Cortés M. T. Motos P. Giménez J. A. (2015). “Consecuencias bio-psico-sociales derivadas del consumo intensivo de alcohol: aspectos psicosociales,” in *Guía Clínica. Consumo Intensivo de Alcohol en Jóvenes*, ed. Cortés (Barcelona: Socidrogalcohol), 95–120.

[B11] Courtney K. E. Polich J. (2009). Binge drinking in young adults: data, definitions, and determinants. *Psychol. Bull.* 135:142. 10.1037/a0014414 19210057 PMC2748736

[B12] Cranford J. A. McCabe S. E. Boyd C. J. (2006). A new measure of binge drinking: prevalence and correlates in a probability sample of undergraduates. *Alcohol. Clin. Exp. Res.* 30 1896–1905. 10.1111/j.1530-0277.2006.00234.x 17067355 PMC1761920

[B13] Crick N. R. Zahn–Waxler C. A. (2003). The development of psychopathology in females and males: current progress and future challenges. *Dev. Psychopathol.* 15 719–742. 10.1017/s095457940300035x 14582938

[B14] De Bruyn S. Wouters E. Ponnet K. Van Damme J. Van Hal G. the Task Force substance use in Flemish universities, . (2017). The psychometric properties of a shortened Dutch version of the consequences scale used in the core alcohol and drug survey. *PLoS One* 12:e0187876. 10.1371/journal.pone.0187876 29216206 PMC5720707

[B15] DeMartini K. S. Carey K. B. (2012). Optimizing the use of the AUDIT for alcohol screening in college students. *Psychol. Assess.* 24:954. 10.1037/a0028519 22612646 PMC3869562

[B16] Devos-Comby L. Lange J. E. (2008). Standardized measures of alcohol-related problems: a review of their use among college students. *Psychol. Addict. Behav.* 22:349. 10.1037/0893-164X.22.3.349 18778128

[B17] Domínguez Lara S. A. (2013). ¿Items politómicos o dicotómicos? Un estudio empírico con una escala unidimensional. *Rev. Argent Cienc. Comport* 5 30–37.

[B18] European Commission (2006). *An EU Strategy to Support Member States in Reducing Alcohol Related Harm.* Brussels: European Commission.

[B19] Falk D. Wang X. Q. Liu L. Fertig J. Mattson M. Ryan M. (2010). Percentage of subjects with no heavy drinking days: evaluation as an efficacy endpoint for alcohol clinical trials. *Alcohol. Clin. Exp. Res*. 34, 2022–2034. 10.1111/j.1530-0277.2010.01290.x 20659066

[B20] Guillamón M. C. Sole A. G. Farran J. C. (1999). Alcohol use disorders identification test (Audit): translation and validation to catalan and Spanish. *Adicciones* 11 337–347.

[B21] Hingson R. White A. (2014). New research findings since the 2007 surgeon general’s call to action to prevent and reduce underage drinking: a review. *J. Stud. Alcohol. Drugs* 75 158–169. 24411808 10.15288/jsad.2014.75.158 PMC3893630

[B22] Hingson R. W. (2010). Magnitude and prevention of college drinking and related problems. *Alcohol. Res. Health* 33 45–54. 23579935 PMC3887494

[B23] Hu L. T. Bentler P. M. (1999). Cutoff criteria for fit indices in covariance structure analysis: conventional criteria versus new alternatives. *Struct. Equ. Modeling* 6 1–55. 10.1080/10705519909540118

[B24] Kahler C. W. Strong D. R. Read J. P. (2005). Toward efficient and comprehensive measurement of the alcohol problems continuum in college students: the brief young adult alcohol consequences questionnaire. *Alcohol. Clin. Exp. Res.* 29 1180–1189. 10.1097/01.alc.0000171940.95813.a5 16046873

[B25] Kahler C. W. Strong D. R. Read J. P. Palfai T. P. Wood M. D. (2004). Mapping the continuum of alcohol problems in college students: a Rasch model analysis. *Psychol. Addict. Behav.* 18 322–333. 10.1037/0893-164X.18.4.322 15631604

[B26] Keough M. T. O’Connor R. M. Read J. P. (2016). Replication and validation of the young adult alcohol consequences questionnaire in a large sample of Canadian undergraduates. *Alcohol. Clin. Exp. Res.* 40 1093–1099. 10.1111/acer.13039 27062148

[B27] Kirouac M. Witkiewitz K. (2018). Revisiting the drinker inventory of consequences: an extensive evaluation of psychometric properties in two alcohol clinical trials. *Psychol. Addict. Behav*. 32, 52–63. 10.1037/adb0000344 29419311 PMC5808585

[B28] Kokotailo P. K. Egan J. Gangnon R. Brown D. Mundt M. Fleming M. (2004). Validity of the alcohol use disorders identification test in college students. *Alcohol. Clin. Exp. Res.* 28 914–920. 10.1097/01.alc.0000128239.87611.f5 15201634

[B29] Kuntsche E. Rehm J. Gmel G. (2004). Characteristics of binge drinkers in Europe. *Soc. Sci. Med.* 59 113–127. 10.1016/j.socscimed.2003.10.009 15087148

[B30] Light L. S. McCoy T. P. Thompson M. P. Spitler H. D. Sutfin E. L. Rhodes S. D. (2011). Modeling the rutgers alcohol problem index (RAPI): a comparison of statistical methods. *Addict. Res. Theory* 19 510–518. 10.3109/16066359.2011.569100

[B31] Lo C. C. (1996). Are women heavier drinkers than we thought they were? *J. Stud. Alcohol.* 57 531–535. 10.15288/jsa.1996.57.531 8858550

[B32] López C. Fernández S. Fernández J. R. Campillo A. Secades R. (2012). Spanish adaptation and validation of the rutgers alcohol problem index (RAPI). *Int. J. Clin. Health Psychol.* 12 251–264.

[B33] López Pina J. A. (2005). Items politómicos vs dicotómicos: un estudio metodológico. *Psicol Spain* 21 339–344.

[B34] Mallett K. A. Varvil-Weld L. Borsari B. Read J. P. Neighbors C. White H. R. (2013). An update of research examining college student alcohol related consequences: new perspectives and implications for interventions. *Alcohol. Clin. Exp. Res*. 37 709–716. 10.1111/acer.12031 23241024 PMC3601564

[B35] Martinez J. A. Sher K. J. Wood P. K. (2014). Drinking consequences and subsequent drinking in college students over 4 years. *Psychol. Addict. Behav.* 28:1240. 10.1037/a0038352 25528051 PMC4708254

[B36] Martinotti G. Lupi M. Carlucci L. Santacroce R. Cinosi E. Acciavatti T. (2017). Alcohol drinking patterns in young people: a survey-based study. *J. Health Psychol.* 22 1889–1896. 10.1177/1359105316667795 27624615 PMC12439245

[B37] Mason-Jones A. J. Cabieses B. (2015). Alcohol, binge drinking and associated mental health problems in young urban Chileans. *PLoS One* 4:e0121116. 10.1371/journal.pone.0121116 25830508 PMC4382020

[B38] Miller W. Tonigan J. S. Longabaugh R. (1995). *The Drinker Inventory of Consequences (DrInC): An Instrument for Assessing Adverse Consequences of Alcohol Abuse*, Vol. 4 Rockville, MD: National Institute on Alcohol Abuse and Alcoholism.

[B39] Motos P. (2013). *Determinantes del Consumo Intensivo de Alcohol en Jóvenes Universitarios.* Ph.D. thesis, Universitat de València, Valencia.

[B40] Moure-Rodríguez L. Caamaño-Isorna F. Doallo S. Juan-Salvadores P. Corral M. Rodríguez-Holguín S. (2014). Heavy drinking and alcohol-related injuries in college students. *Gac. Sanit.* 28 376–380. 10.1016/j.aap.2016.12.012 24725631

[B41] National Institute on Alcohol Abuse and Alcoholism [NIAAA] (2004). *NIAAA Council Approves Definition of Binge Drinking. NIAAA Newsletter, 3.* Available online at: https://pubs.niaaa.nih.gov/publications/Newsletter/winter2004/Newsletter_Number3.pdf (accessed March 29, 2004).

[B42] National Institute on Alcohol Abuse and Alcoholism [NIAAA (2019). *Fall Semester-A Time for Parents to Discuss the Risks of College Drinking*. Available online at: https://www.niaaa.nih.gov/sites/default/files/publications/NIAAA_BacktoCollege_Fact_sheet.pdf

[B43] Neal D. J. Corbin W. R. Fromme K. (2006). Measurement of alcohol-related consequences among high school and college students: application of item response models to the rutgers alcohol problem index. *Psychol. Assess.* 18 402–414. 10.1037/1040-3590.18.4.402 17154761

[B44] Norman P. Conner M. T. Stride C. B. (2012). Reasons for binge drinking among undergraduate students: an application of behavioural reasoning theory. *Br. J. Health Psychol.* 17 682–698. 10.1111/j.2044-8287.2012.02065.x 22420300

[B45] Observatorio Español de las Drogas y las Adicciones [OEDA] (2017). *Encuesta Sobre Alcohol y Otras Drogas En España (EDADES).* Madrid: Ministerio De Sanidad, Consumo y Bienestar Social.

[B46] Parada M. Corral M. Caamañ;o-Isorna F. Mota N. Crego A. Holguín S. R. (2011). Definición del concepto de consumo intensivo de alcohol adolescente (binge drinking). *Adicciones* 23 53–63. 10.20882/adicciones.167 21503564

[B47] Perkins H. W. (2002). Surveying the damage: a review of research on consequences of alcohol misuse in college populations. *J. Stud. Alcohol.* Supplement 91–100. 10.15288/jsas.2002.s14.91 12022733

[B48] Pilatti A. Read J. P. Caneto F. (2016). Validation of the Spanish version of the young adult alcohol consequences questionnaire (S-YAACQ). *Psychol. Assess.* 28:e49. 10.1037/pas0000140 26302103

[B49] Pilatti A. Read J. P. Pautassi R. M. (2017). ELSA 2016 cohort: alcohol, tobacco, and marijuana use and their association with age of drug use onset, risk perception, and social norms in Argentinean college freshmen. *Front. Psychol.* 8:e1452. 10.3389/fpsyg.2017.01452 28890707 PMC5575425

[B50] Pilatti A. Read J. P. Vera B. D. Caneto F. Garimaldi J. A. Kahler C. W. (2014). The Spanish version of the brief young adult alcohol consequences questionnaire (B-YAACQ): a Rasch model analysis. *Addict. Behav.* 39 842–847. 10.1016/j.addbeh.2014.01.026 24583273

[B51] Read J. P. Haas A. L. Radomski S. Wickham R. E. Borish S. E. (2016). Identification of hazardous drinking with the young adult alcohol consequences questionnaire: relative operating characteristics as a function of gender. *Psychol. Assess.* 28 1276–1289. 10.1037/pas0000251 26691503

[B52] Read J. P. Kahler C. W. Strong D. R. Colder C. R. (2006). Development and preliminary validation of the young adult alcohol consequences questionnaire. *J. Stud. Alcohol* 67 169–177. 10.15288/jsa.2006.67.169 16536141

[B53] Read J. P. Merrill J. E. Kahler C. W. Strong D. R. (2007). Predicting functional outcomes among college drinkers: reliability and predictive validity of the young adult alcohol consequences questionnaire. *Addict. Behav.* 32 2597–2610. 10.1016/j.addbeh.2007.06.021 17706888

[B54] Rosón H. B. (2008). Consumo de riesgo y perjudicial de alcohol. Prevalencia y métodos de detección en la práctica clínica. *Galicia Clínica* 69 29–44.

[B55] Scully K. A. Mohn R. S. Madson M. B. (2018). Psychometric evaluation of the drinking refusal self-efficacy scale – revised with college students in the United States. *Addict. Behav.* 85 100–106. 10.1016/j.addbeh.2018.05.032 29883855

[B56] Slutske W. S. (2005). Alcohol use disorders among US college students and their non–college-attending peers. *Arch. Gen. Psychiatry* 62 321–327. 10.1001/archpsyc.62.3.321 15753245

[B57] Sobell L. C. Sobell M. B. (1996). *Timeline FollowBack: User’s Guide.* Ontario: Addiction Research Foundation.

[B58] Substance Abuse and Mental Health Services Administration [SAMHSA] (2016). *Results from the 2015 National Survey on Drug Use and Health.* Rockville, MD: Center for Behavioral Health Statistics and Quality.

[B59] Townshend J. M. Duka T. (2005). Binge drinking, cognitive performance and mood in a population of young social drinkers. *Alcohol. Clin. Exp. Res.* 29 317–325. 10.1097/01.alc.0000156453.05028.f5 15770105

[B60] Van Dam N. T. Earleywine M. Borders A. (2010). Measuring mindfulness? an item response theory analysis of the mindful attention awareness scale. *Pers. Individ. Dif.* 49 805–810. 10.1016/j.paid.2010.07.020

[B61] Wang M. Russell S. S. (2005). Measurement equivalence of the job descriptive index across Chinese and American workers: results from confirmatory factor analysis and item response theory. *Educ. Psychol. Meas.* 65 709–732. 10.1177/0013164404272494

[B62] Wechsler H. Davenport A. Dowdall G. Moeykens B. Castillo S. (1994). Health and behavioral consequences of binge-drinking in college. a national survey of students at 140 campuses. *JAMA* 272 1672–1677. 10.1001/jama.272.21.1672 7966895

[B63] Wechsler H. Nelson T. F. (2008). What we have learned from the Harvard school of public health college alcohol study: focusing attention on college student alcohol consumption and the environmental conditions that promote it. *J. Stud. Alcohol. Drugs.* 69 481–490. 10.15288/jsad.2008.69.481 18612562

[B64] White H. R. Labouvie E. W. (1989). Towards the assessment of adolescent problem drinking. *J. Stud. Alcohol.* 50 30–37. 10.15288/jsa.1989.50.30 2927120

[B65] White H. R. McMorris B. J. Catalano R. F. Fleming C. B. Haggerty K. P. Abbott R. D. (2006). Increases in alcohol and marijuana use during the transition out of high school into emerging adulthood: the effects of leaving home, going to college, and high school protective factors. *J. Stud. Alcohol.* 67 810–822. 10.15288/jsa.2006.67.810 17060997 PMC2314672

[B66] World Health Organization [WHO] (2001). *AUDIT: The Alcohol Use Disorders Identification Test: Guidelines for Use in Primary Health Care*, 2nd Edn Geneve: World Health Organization.

[B67] World Health Organization [WHO] (2007). *WHO Expert Committee on Problems Related to Alcohol Consumption: Second Report (Sep 19).* Brussels: World Health Organization. 17970166

[B68] World Health Organization [WHO] (2018). *Global Status Report on Alcohol and Health 2018.* Geneva: World Health Organization.

